# Myeloid sarcoma masquerading as central nervous system diffuse large b-cell lymphoma: a case report and literature review

**DOI:** 10.3389/fonc.2025.1712803

**Published:** 2026-01-05

**Authors:** Wanli Hu, Yang Liu, Chengcheng Ma, Yuexian Gao, Pengyun Zeng, Huiling Chen

**Affiliations:** Department of Hematology, Lanzhou University Second Hospital, Lanzhou University, Lanzhou, China

**Keywords:** acute myeloid leukemia, central nervous system, diffuse large B-cell lymphoma, masquerade, myeloid sarcoma, RUNX1::RUNX1T1

## Abstract

This report presents a challenging case of a 52-year-old male with a history of acute myeloid leukemia (AML) with t(8;21)(q22;q22.1)/RUNX1::RUNX1T1 who developed an intracranial mass during hematologic remission. Initial histopathological examination of the resected lesion, due to aberrant expression of B-cell markers (PAX-5, CD79a, c-Myc, Bcl-2), led to a misdiagnosis of diffuse large B-cell lymphoma (DLBCL). Subsequent comprehensive integration of clinical history, repeated bone marrow assessment, cytogenetics, fluorescence *in situ* hybridization (FISH), and extended immunohistochemistry(IHC) revealed the tumor to be a myeloid sarcoma (MS), representing an extramedullary relapse of his underlying AML. This case underscores the diagnostic pitfalls of MS, particularly within the central nervous system (CNS), and highlights the critical importance of considering MS in patients with a history of AML, especially those with genetic profiles predisposing to extramedullary disease, even when pathology initially suggests lymphoma.

## Case presentation

A 52-year-old male was initially admitted to a local hospital on June 5, 2022, presenting with one-month history of dizziness and fatigue, worsening over the preceding week. Laboratory investigations revealed leukocytosis (WBC 35.73×10^9^/L), severe anemia (HGB 48 g/L), profound thrombocytopenia (PLT 7×10^9^/L), and elevated lactate dehydrogenase (757.94 U/L). Bone marrow morphology showed hypercellular marrow with 78.5% blasts, positive for myeloperoxidase staining, consistent with acute myeloid leukemia with maturation. Immunophenotyping identified 54.9% blasts expressing myeloid antigens along with CD19 and CD56. Cytogenetic analysis revealed the karyotype 46, XY, t(8;21)(q22;q22.1). Molecular testing was positive for the RUNX1::RUNX1T1 fusion gene (338.47%) and negative for FLT3, NPM1, and KIT mutations. A definitive diagnosis of AML with RUNX1::RUNX1T1 was established. At initial diagnosis, only a CT of the chest and an abdominal ultrasound were performed, both of which were unremarkable. No other imaging studies were conducted. The patient achieved remission following induction chemotherapy with the IA regimen (Idarubicin 15 mg d1-3, Cytarabine 200 mg d1-7).

On July 21, 2022, the patient was readmitted for a follow-up bone marrow measurable residual disease (MRD) test. The test revealed detectable MRD at a level of 1.6×10^−4^ by flow cytometry(FCM; sensitivity 5×10^−5^, a decreased RUNX1::RUNX1T1 copy number of 1.89%(via RT-qPCR, sensitivity 1×10-4), and a normal karyotype, prompting consolidation chemotherapy with the IA regimen again. However, upon reevaluation on September 1, 2022, detectable bone marrow MRD by FCM increased to 6.6×10^−4^ and the RUNX1::RUNX1T1 copy number rose to 4.57%, leading to treatment with venetoclax combined with the HA regimen(Venetoclax 200mg d1-14, Homoharringtonine 1.6mg d1-10, Cytarabine 32mg d1-10). A cranial CT scan obtained during this admission was unremarkable. During subsequent admissions on November 19, 2022, and February 22, 2023, detectable FCM-based bone marrow MRD levels were 3.3×10^−4^ and 3.0×10^−4^, respectively, with continued venetoclax-based consolidation therapy. The final admission (October 14, 2023) demonstrated a detectable bone marrow MRD level of 1.4×10^−4^ by FCM, with the RUNX1::RUNX1T1 transcript measured at 0.06%, the patient was prescribed daily venetoclax but subsequently did not adhere to regular treatment. Throughout this period, bone marrow morphology consistently indicated complete remission. Intrathecal chemotherapy (methotrexate, cytarabine, dexamethasone) was administered on two separate occasions, during the second and fourth hospital admissions. The patient’s clinical course was marked by poor tolerance to chemotherapy, with multiple episodes of myelosuppression, resulting in low motivation for further intensive treatment. Consequently, despite the clinical recommendation for allogeneic hematopoietic stem cell transplantation (allo-HSCT) due to persistent detectable MRD, the patient declined this option for personal reasons.

**Figure 1:**
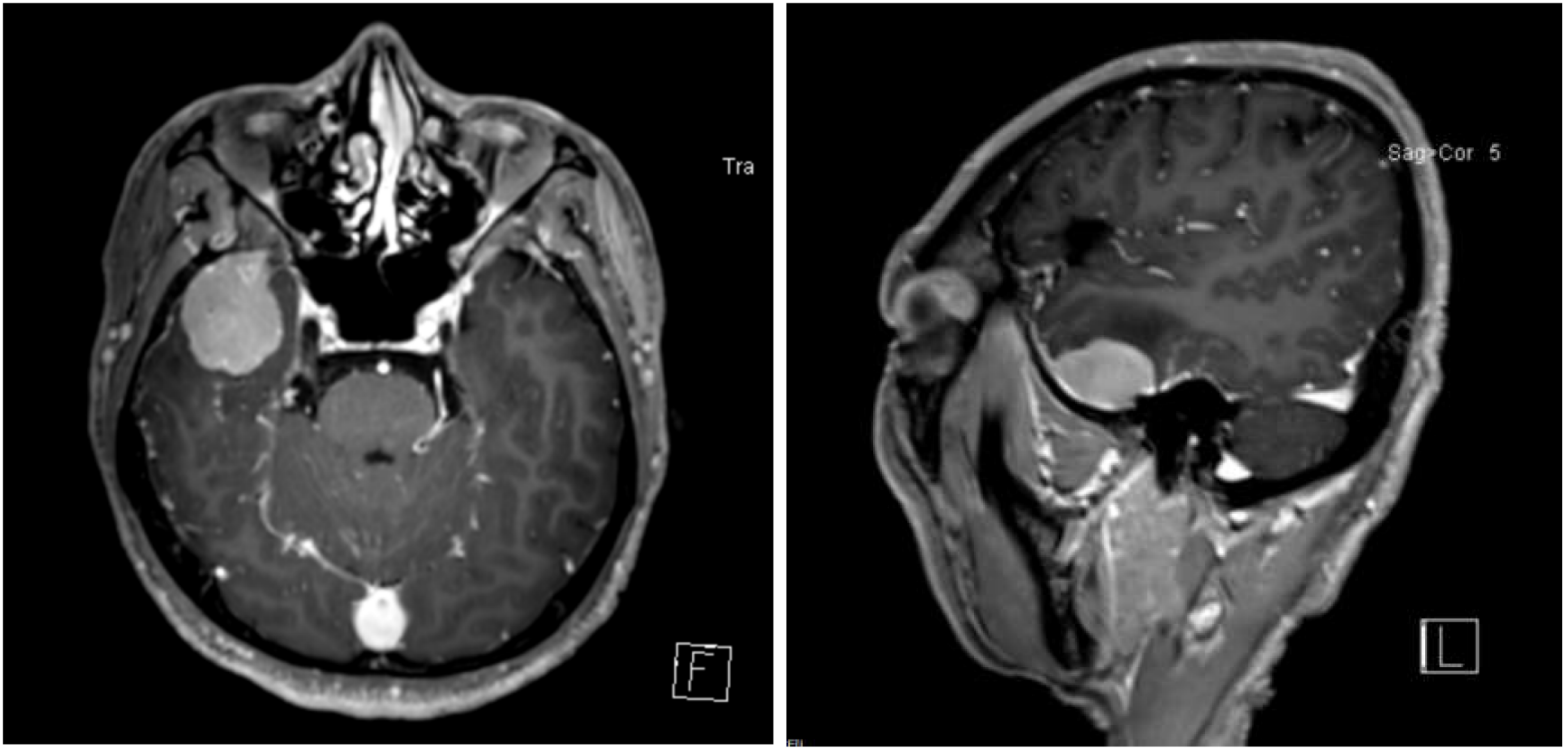
Contrast-enhanced cranial MRI demonstrating a space-occupying lesion in the right temporal region.

**Figure 2:**
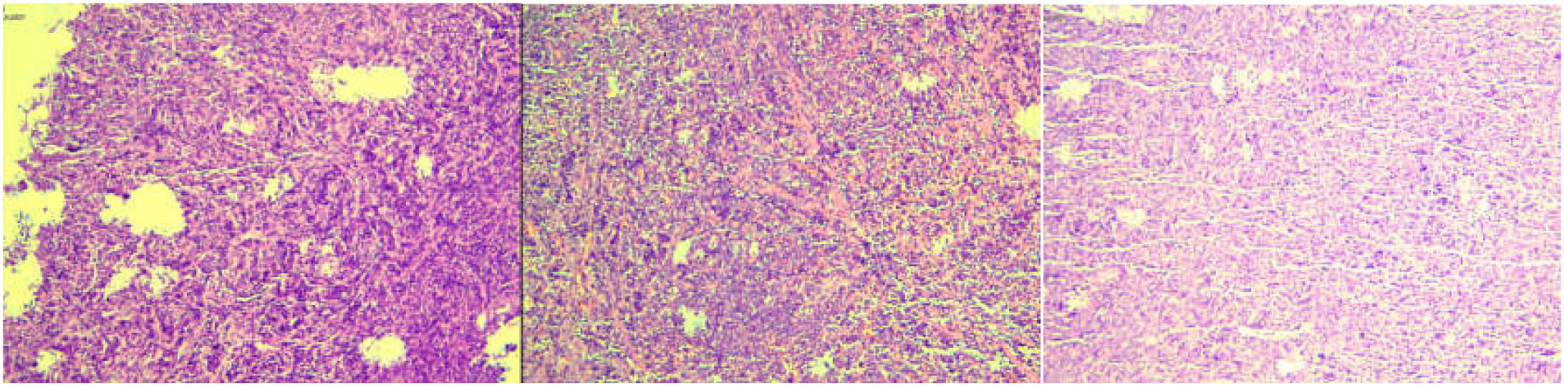
Pathological characteristics of the intracranial lesion.

On December 17, 2024, he presented to our neurosurgery department with a 15-day history of dizziness. Blood counts were within normal limits. Cranial contrast-enhanced MRI revealed a well-defined, round, mass-like lesion (approx. 28.2×36.3×22.4 mm) in the right temporal region. The lesion showed homogeneous enhancement, a broad dural base with a dural tail sign, and minimal perilesional edema, radiologically suggestive of meningioma (**Figure 1**). The patient underwent lesion resection. Initial histopathological examination of the intracranial lesion suggested non-Hodgkin lymphoma, diffuse large B-cell type, of non-germinal center origin, with IHC indicating c-Myc and Bcl-2 double expression. IHC results were: CD79a(+), PAX-5(+), CD10(-), Bcl-2(90%+), Bcl-6(-), c-Myc(40%+), Ki-67(90%+). EBER-ISH was negative. FISH for MYC and BCL2 rearrangements was negative (**Figure 2**).

He was transferred to our hematology unit on January 31, 2025, with a referral diagnosis of DLBCL. Physical examination was unremarkable. CBC showed WBC 1.26×10^9^/L, monocyte percentage 38.9%, PLT 25×10^9^/L. PET-CT revealed hypermetabolic soft tissue thickening in the right temporal surgical bed (SUVmax 11.1), along with metabolically active nodules in the left lateral ventricle, right cerebellar peduncle, and sacral canal (SUVmax 8.4). Diffuse mild metabolic increase was observed in the central bone marrow (SUVmax 3.6), with no other hypermetabolic foci detected. Genetic testing at CNS relapse on the intracranial mass specimen identified a truncating mutation in the MGA gene (p.R312Ter, VAF 45.33%; p.K2092Ter, VAF 38.47%). A provisional diagnosis of non-GCB DLBCL (Stage IV, IPI 2) was made, and treatment with R-MTX (Rituximab 600mg d1, Methotrexate 6g d2) was commenced.

**Figure 3:**
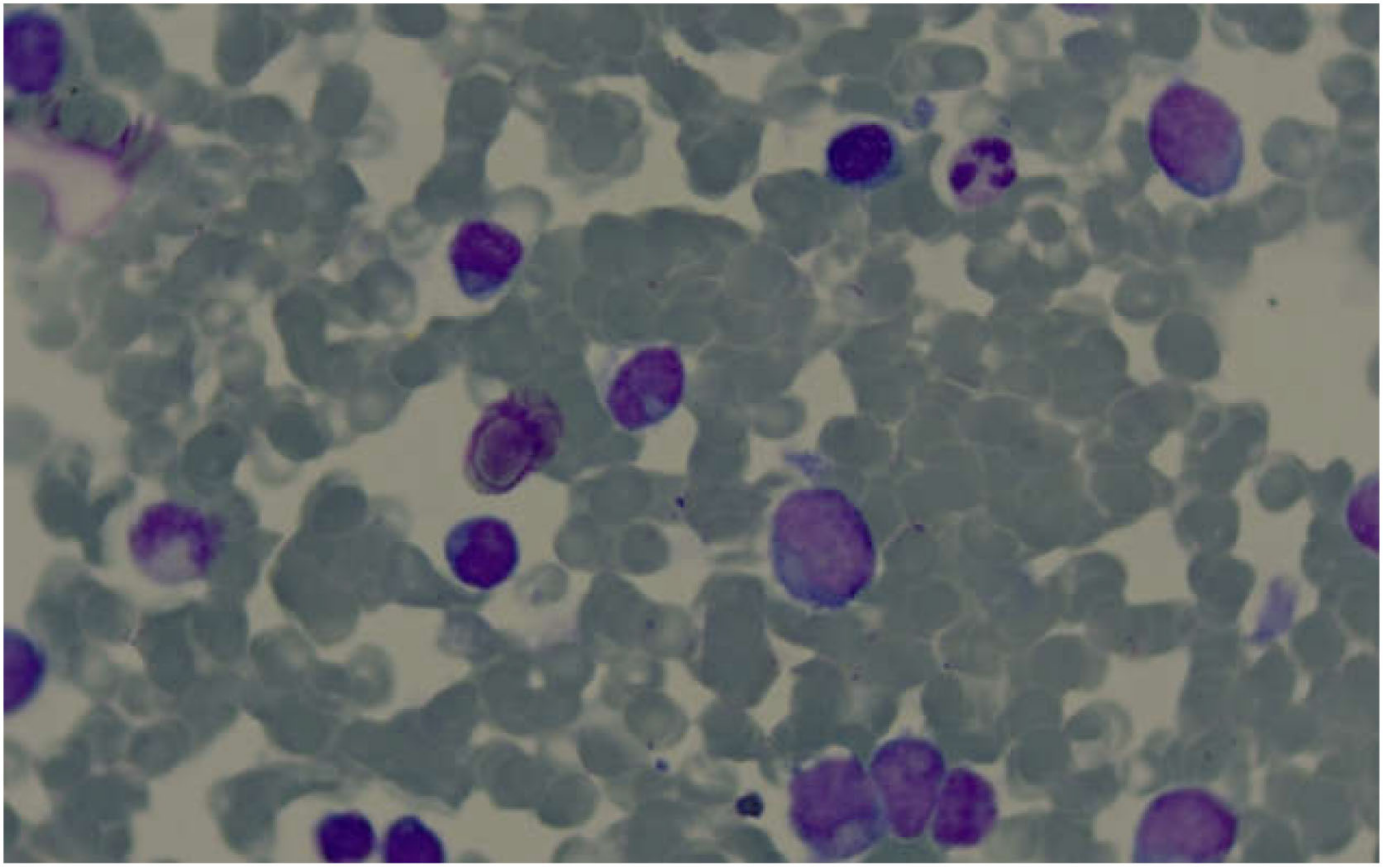
Bone marrow cytomorphology displaying a large number of primitive cells. .

**Figure 4:**
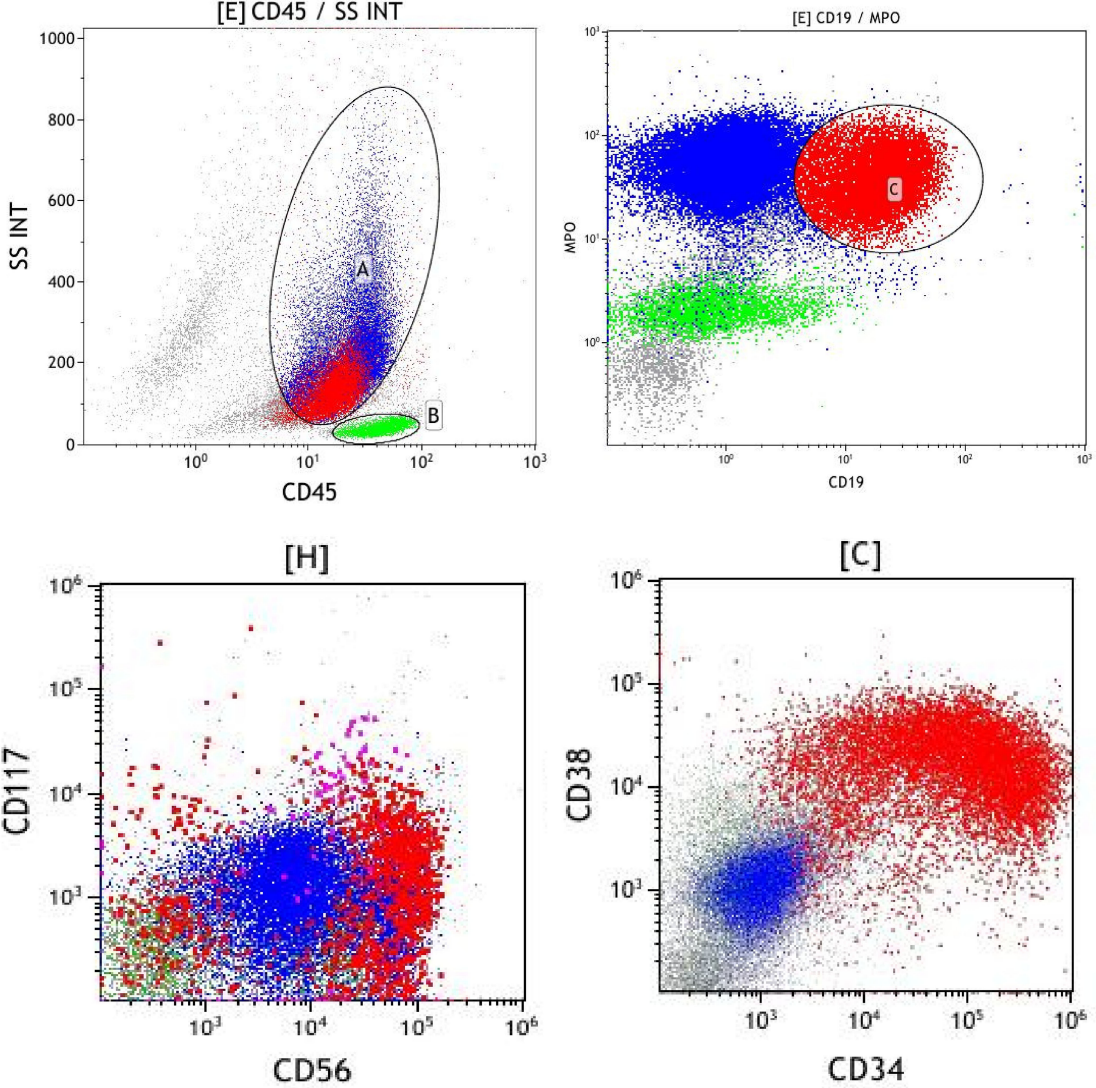
Bone marrow FCM indicating that the immature cell population expresses CD34 and MPO, with abnormal expression of CD19 and CD56.

**Figure 5:**
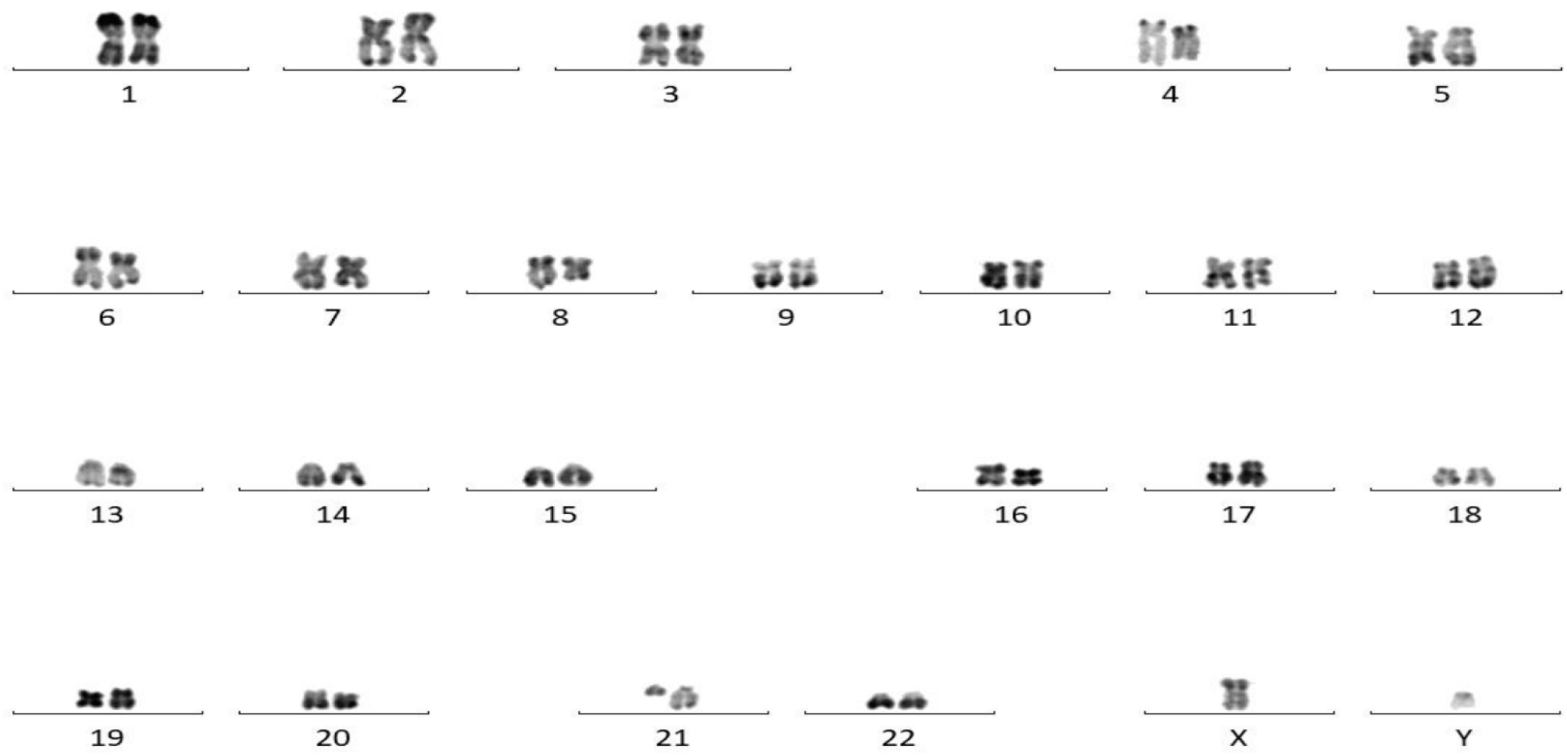
Karyotype analysis demonstrating t(8;21).

**Figure 6:**
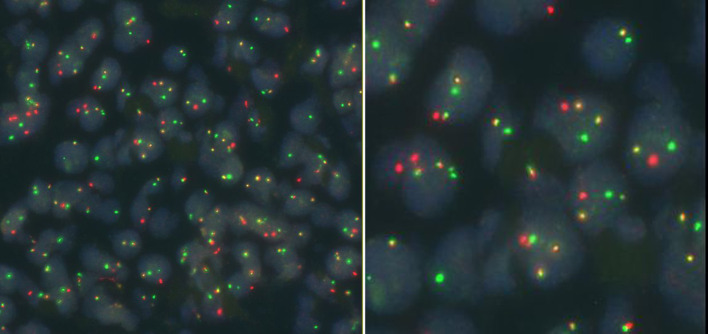
FISH testing of the intracranial lesion detecting ETO/AML1 fusion.

The bone marrow results became available after chemotherapy. Bone marrow biopsy revealed the presence of tumor cells, and immunohistochemical staining showed that the tumor cells were positive for CD34, MPO, and CD99, and weakly positive for PAX-5, suggesting a diagnosis of AML. Bone marrow cytomorphology showed markedly active bone marrow hyperplasia, with primitive granulocytes accounting for 28% and immature monocytes accounting for 2%, indicating AML relapse (**Figure 3**). Bone marrow FCM confirmed 21.3% blasts with myeloid antigen expression and aberrant CD19/CD56 (**Figure 4**). Karyotyping again showed 46, XY, t(8;21)(q22;q22) (10) (**Figure 5**). RUNX1::RUNX1T1 fusion was positive. Mutation profiling identified CSF3R (34.69%), DNMT3A (40.76%), SMC3 (1.11%), and ZBTB7A (39.85%) mutations. Initially, the patient was suspected to have a relapse of AML with a concurrent second malignancy, central nervous system DLBCL. Given the patient’s poor performance status, this clinical impression guided our decision to initiate therapy with Venetoclax and Azacitidine, targeting the suspected relapsed AML. To determine the clonal relationship between the intracranial lesion and the AML, and prompted by the unusual presentation of ‘dual malignancies’, FISH analysis was performed on the archived brain tissue using ETO/AML1 probes, which confirmed the fusion signal (86%) (**Figure 6**). Additional IHC on the brain tissue showed CD43(+), MPO(focal+), CD117(weak+), and CD20(-), contradicting the initial DLBCL diagnosis. The findings were consistent with myeloid sarcoma exhibiting aberrant B-cell marker expression. The diagnosis was revised to intracranial myeloid sarcoma, representing extramedullary relapse of AML (**Table 1**). Consequently, the initial therapeutic regimens of R-MTX and Venetoclax plus Azacitidine, which were instituted based on the initial diagnostic impression, proved to be inappropriate in light of the revised diagnosis.

**Table 1:** Summary of key diagnostic findings at different time points.

Time point	Immunophenotype (FCM/IHC)	Cytogenetics/FISH	Molecular findings (PCR/NGS)
Initial AML Diagnosis (Jun 2022)	Myeloid blasts (MPO+), aberrant CD19/CD56	BM:46, XY, t(8;21)(q22;q22.1)	BM: RUNX1::RUNX1T1 fusion positive
CNS Relapse (Dec 2024) (Initial Misdiagnosis)	CD79a(+), PAX-5(+), c-Myc(40%+), Bcl-2(90%+), Ki-67(90%+)	Intracranial Mass: FISH for MYC/BCL2 rearrangements: Negative	Not performed at this time
CNS Relapse (Dec 2024) (Corrected Diagnosis)	CD43(+), MPO(focal+), CD117(weak+), CD20(-)	Intracranial Mass: FISH for ETO/AML1 fusion: positive	Intracranial Mass: *MGA* p.R312Ter (VAF 45.33%); p.K2092Ter (VAF 38.47%)
BM Relapse (Jan 2025)	Blasts (21.3%) positive for CD34, MPO, aberrant CD19/CD56	BM:46, XY, t(8;21)(q22;q22)	BM: RUNX1::RUNX1T1 fusion positive. *CSF3R* (VAF 34.69%), *DNMT3A* (VAF 40.76%), *SMC3* (VAF 1.11%), *ZBTB7A* (VAF 39.85%)

During hospitalization, the patient’s neurological status deteriorated. Follow-up MRI indicated disease progression (Patchy areas within the right middle cerebellar peduncle show mild T1WI and T2WI prolongation with mild hyperintensity on T2WI/FLAIR and demonstrate marked enhancement. Additional numerous punctate enhancing foci are noted in the pons and bilateral cerebellar hemispheres). The family opted for palliative care and discharge. The patient was subsequently discharged in a deteriorating condition and was lost to follow-up.

## Discussion

This case involves a 52-year-old male who was initially diagnosed with typical AML with RUNX1::RUNX1T1 fusion. The patient achieved morphologic leukemia-free state following multiple rounds of chemotherapy, however, MRD remained detectable. In accordance with the ELN 2022 guidelines, persistent detectable MRD in RUNX1::RUNX1T1-positive AML generally necessitates treatment escalation and consideration of allo-HSCT in eligible patients (1). Although this recommendation was made, the patient’s decision to decline was based on personal preference. One year after discontinuing treatment, the patient developed an intracranial space-occupying lesion. Due to the abnormal expression of certain B-cell antigens (such as PAX-5+, CD79a+) by the tumor cells, the initial pathological report incorrectly diagnosed the lesion as “diffuse large B-cell lymphoma”. Subsequent comprehensive integration of molecular genetic testing, immunophenotypic analysis, and bone marrow reexamination results led to a revised diagnosis of myeloid sarcoma (MS). This case underscores the diagnostic complexity of MS, particularly CNS-MS, and the significant challenges posed by its morphological similarity to lymphoma (2, 3).

The development of myeloid sarcoma is closely linked to specific genetic subtypes of AML. AML with t(8;21)(q22;q22.1) translocation, which forms the RUNX1::RUNX1T1 fusion gene, is one of the known subtypes with the highest risk of concurrent MS (4). The patient in this case was initially diagnosed with this subtype, which laid a critical biological foundation for the subsequent development of MS. Beyond t(8;21), AML with inv(16)(p13.1q22) or t(16;16)(p13.1;q22) translocations (forming the CBFB::MYH11 fusion gene), AML with monocytic differentiation, and AML with MLL (KMT2A) gene rearrangement, AML with expression of CD56 have all been shown to have a relatively high risk of developing MS (5–7). Therefore, for AML patients with the aforementioned genetic backgrounds, even when in hematological remission, clinicians should maintain a high level of vigilance for extramedullary lesions (8). It is important to note that while these subtypes are high-risk factors for MS, the overall prevalence of MS remains low (2), and cases of primary or isolated relapse in the CNS are even rarer (9, 10), his rarity results in clinicians having limited awareness and clinical experience regarding this condition.

The central nervous system, as an “immune-privileged” site, is a relatively uncommon but highly challenging location for MS involvement (11). CNS-MS can present as an isolated mass or occur simultaneously or sequentially with bone marrow relapse (12). Its clinical manifestations are varied and non-specific, often causing symptoms such as headache, vomiting, focal neurological deficits, epilepsy, or consciousness disturbance due to mass effect. This makes it easily confused with primary central nervous system lymphoma (PCNSL), meningioma, metastatic tumors, or infectious diseases. On MRI sequences, CNS-MS further manifests with isointense/hypointense signals on T1WI, hyperintense signals on T2WI/FLAIR, restricted diffusion on DWI, and present as dural-based enhancing lesions, often accompanied by a “dural tail sign”—all of which align with the MRI findings in this case. In contrast, central nervous system lymphoma usually shows uniform enhancement within the brain parenchyma and may also exhibit a dural tail sign (predominantly leptomeningeal tail sign) as well as restricted diffusion on DWI; however, it mostly presents with isointense/hypointense signals on T2WI, a feature distinguishing it from CNS-MS (8, 13–15). Although there are differences between the two, there is considerable overlap, which is insufficient to serve as a reliable basis for differentiation (11). Studies indicate that CNS-MS and suspected lymphoma share similar hypermetabolic features on PET-CT to the extent that they cannot be reliably differentiated based on metabolic characteristics alone (16). The gold standard for diagnosis still relies on pathological examination; however, as illustrated in this case, routine biopsy specimens are highly prone to misdiagnosis. The prognosis of CNS-MS is extremely poor, and the presence of the blood-brain barrier makes it difficult for most chemotherapeutic drugs to reach effective therapeutic concentrations, creating a major barrier to clinical treatment (11, 17).

In this case, the tumor cells expressed CD79a and PAX-5, along with high expression of c-Myc and Bcl-2, which closely resembled the characteristics of “double-expressor DLBCL” in terms of pathological morphology (18). This cross-lineage expression phenomenon has been previously reported in AML with t(8;21). Such tumor cells typically express CD19 and CD56 abnormally and may occasionally express B-cell markers (19–21). Studies have indicated that the RUNX1::RUNX1T1 fusion protein may regulate the expression of genes involved in hematopoietic cell differentiation, leading to the abnormal expression of lymphoid markers such as CD19, CD79a, and PAX-5 in myeloid blasts. This mechanism has been partially confirmed in t(8;21)-positive AML (21, 22).Notably, the supplementary immunohistochemical results of the intracranial lesion in this case showed CD20 negativity and MPO positivity, which provided crucial evidence for ultimately ruling out B-cell lymphoma and confirming the diagnosis of myeloid sarcoma (2).

In-depth molecular testing ultimately uncovered the true nature of the lesion. FISH confirmed the presence of the ETO/AML1 fusion signal in the intracranial lesion, suggesting homology with the primary AML (2).Molecular biological testing not only confirmed the presence of the RUNX1::RUNX1T1 fusion gene, which was identical to that in the initially diagnosed AML, but also identified truncating mutations in the MGA gene. As an important tumor suppressor gene, inactivating mutations of MGA are common in lymphoma and may lead to high expression of c-Myc by releasing the inhibition of the MYC signaling pathway. MGA truncating mutations are also frequently observed in CBF-AMLs and have been shown to cooperate with RUNX1::RUNX1T1 to accelerate leukemogenesis in preclinical models, supporting a tumor-suppressor role (23).Studies have confirmed that in mouse hematopoietic cells with MGA deletion, the expression of RUNX1::RUNX1T1 results in more aggressive AML with a significantly shortened latency period (24). It should be acknowledged that current data on MGA as an independent adverse prognostic factor in RUNX1::RUNX1T1 AML are limited, and the association observed in this case is hypothesis-generating rather than definitive. Risk assessment should integrate co-mutations, MRD kinetics, and clinical features including CNS involvement. In addition, the DNMT3A, CSF3R, and ZBTB7A mutations detected in this case may collectively form the molecular basis for the high aggressiveness of the disease. The DNMT3A mutation is closely associated with treatment resistance and clonal evolution (25).The CSF3R mutation can activate the JAK-STAT pathway (26), and it is hypothesized that this may enhance the sensitivity of leukemia cells to factors such as G-CSF in the central nervous system microenvironment, thereby promoting their migration to and survival within the CNS (27), ZBTB7A, as a transcriptional repressor and potential tumor suppressor gene, its mutation may promote cell proliferation and impair differentiation ability by releasing the inhibition of multiple target genes. The synergistic effect of these mutation with the RUNX1::RUNX1T1 fusion protein has been confirmed (28), The synergistic effects of multiple functional mutations in epigenetic modification, signal pathway activation, and other aspects may be the underlying reasons for the complex clinical manifestations and difficult treatment of this case.

The optimal management of isolated CNS relapse of AML or MS remains challenging due to its rarity. Systemic therapy with high CNS penetration, such as high-dose cytarabine (HiDAC), forms the cornerstone of treatment. This can be combined with CNS-directed radiotherapy for bulky disease (29). The role of intrathecal chemotherapy is also considered, though its efficacy alone is limited (6). For eligible patients who achieve a response, consolidation with allo-HSCT should be strongly considered due to the high risk of subsequent systemic relapse. Regarding CNS prophylaxis, it is typically reserved for AML with a recognized high risk of CNS involvement (6, 29).

In summary, this case serves as a critical reminder for clinicians and pathologists. In patients with a history of AML, especially those with genetic backgrounds prone to extramedullary infiltration such as t(8;21), if new extramedullary masses appear, even if the initial pathology suggests lymphoma, the possibility of myeloid sarcoma must be highly suspected. The final diagnosis must be based on a comprehensive integration of clinical history, morphological characteristics, immunophenotype (requiring a complete set of myeloid and lymphoid antibody panels), cytogenetics, and molecular biology results. Avoiding misdiagnosis is a critical step in implementing appropriate treatment and improving patient prognosis.

## Data Availability

The original contributions presented in the study are included in the article, further inquiries can be directed to the corresponding authors.

## References

[1] Döhner H Wei AH Appelbaum FR Craddock C DiNardo CD Dombret H . Diagnosis and management of AML in adults: 2022 recommendations from an international expert panel on behalf of the ELN. Blood. (2022) 140:1345–77. doi: 10.1182/blood.2022016867, PMID: 35797463

[2] Patkowska E Krzywdzinska A Solarska I Wojtas M Prochorec-Sobieszek M . Diagnostic approaches in myeloid sarcoma. Curr Issues Mol Biol. (2025) 47:111. doi: 10.3390/cimb47020111, PMID: 39996833 PMC11853749

[3] Musleh M Musleh S Sheikhi M . Myeloid sarcoma in brain and optic nerve presented as a relapse of acute myeloid leukemia: A case report. Clin Case Rep. (2024) 12:e8861. doi: 10.1002/ccr3.8861, PMID: 38721563 PMC11077169

[4] Chen SW Li CF Chuang SS Tzeng CC Hsieh YC Lee PS . Aberrant co-expression of CD19 and CD56 as surrogate markers of acute myeloid leukemias with t(8;21) in Taiwan. Int J Lab Hematol. (2008) 30:133–8. doi: 10.1111/j.1751-553X.2007.00913.x, PMID: 18333845

[5] Pileri SA Ascani S Cox MC Campidelli C Bacci F Piccioli M . Myeloid sarcoma: clinico-pathologic, phenotypic and cytogenetic analysis of 92 adult patients. Leukemia. (2007) 21:340–50. doi: 10.1038/sj.leu.2404491, PMID: 17170724

[6] Siegal T Benouaich-Amiel A Bairey O . Neurologic complications of acute myeloid leukemia. Diagn approach Ther modalities. Blood Rev. (2022) 53:100910. doi: 10.1016/j.blre.2021.100910, PMID: 34836656

[7] Diamantidis MD . Myeloid sarcoma: novel advances regarding molecular pathogenesis, presentation and therapeutic options. J Clin Med. (2024) 13:6154. doi: 10.3390/jcm13206154, PMID: 39458104 PMC11509401

[8] Cuglievan B Menegaz BA Garces S Rytting ME . Acute myeloid leukaemia masquerading as a primary CNS tumour. BMJ Case Rep. (2017) 2017:bcr2017220891. doi: 10.1136/bcr-2017-220891, PMID: 28801332 PMC5623295

[9] Cheng H Di G Gao W Chenhui Z Jiang X . A clinical report of intracranial granulocytic sarcoma and a literature review. Int J Neurosci. (2022) 132:945–9. doi: 10.1080/00207454.2020.1858824, PMID: 33272089

[10] Cervantes GM Cayci Z . Intracranial CNS manifestations of myeloid sarcoma in patients with acute myeloid leukemia: review of the literature and three case reports from the author’s institution. J Clin Med. (2015) 4:1102–12. doi: 10.3390/jcm4051102, PMID: 26239467 PMC4470219

[11] Olar A Lapadat R Davidson CJ Stein TD Dahiya S Perry A . Central nervous system involvement by myeloid sarcoma: a report of 12 cases and review of the literature. Clin Neuropathol. (2016) 35:314–25. doi: 10.5414/NP300949, PMID: 27125868

[12] Kaur V Swami A Alapat D Abdallah AO Motwani P Hutchins LF . Clinical characteristics, molecular profile and outcomes of myeloid sarcoma: a single institution experience over 13 years. Hematology. (2018) 23:17–24. doi: 10.1080/10245332.2017.1333275, PMID: 28574302

[13] Chaudhry AA Gul M Chaudhry AA Dunkin J . Qualitative assessment of diffusion weighted imaging and susceptibility weighted imaging of myeloid sarcoma involving the brain. J Comput Assist Tomogr. (2016) 40:61–6. doi: 10.1097/RCT.0000000000000337, PMID: 26599963

[14] Yang B Yang C Fang J Yang J Xu Y . Clinicoradiological characteristics, management and prognosis of primary myeloid sarcoma of the central nervous system: A report of four cases. Oncol Lett. (2017) 14:3825–31. doi: 10.3892/ol.2017.6620, PMID: 28927153 PMC5587933

[15] Lasocki A Seymour JF . Central nervous system manifestations of systemic haematological Malignancies and key differentials. Clin Radiol. (2022) 77:328–36. doi: 10.1016/j.crad.2022.01.043, PMID: 35164931

[16] Kei PL Kok TY Linn YC Padhy AK . Butterfly lesion of the corpus callosum: an unusual case of extramedullary myeloid sarcoma (granulocytic sarcoma). Clin Nucl Med. (2011) 36:365–6. doi: 10.1097/RLU.0b013e31820aa1b4, PMID: 21467855

[17] Kohli V Koltz MT Kamath AA . Isolated recurrence of acute myeloid leukemia in the cerebellum: illustrative case. J Neurosurg Case Lessons. (2021) 2:Case21281. doi: 10.3171/CASE21281, PMID: 35855087 PMC9265191

[18] Ananthamurthy A . An immunohistochemical study of double-expressor lymphomas and its correlation with cell of origin. J Cancer Res Ther. (2023) 19:S0. doi: 10.4103/jcrt.jcrt_587_21, PMID: 37147953

[19] Haferlach T Meggendorfer M . More than a fusion gene: the RUNX1-RUNX1T1 AML. Blood. (2019) 133:1006–7. doi: 10.1182/blood-2019-01-896076, PMID: 30846508

[20] Tiacci E Pileri S Orleth A Pacini R Tabarrini A Frenguelli F . PAX5 expression in acute leukemias: higher B-lineage specificity than CD79a and selective association with t(8;21)-acute myelogenous leukemia. Cancer Res. (2004) 64:7399–404. doi: 10.1158/0008-5472.CAN-04-1865, PMID: 15492262

[21] Valbuena JR Medeiros LJ Rassidakis GZ Hao S Wu CD Chen L . Expression of B cell-specific activator protein/PAX5 in acute myeloid leukemia with t(8;21)(q22;q22). Am J Clin Pathol. (2006) 126:235–40. doi: 10.1309/LG0Q0VXYBETJ4VHE, PMID: 16891199

[22] Al-Harbi S Aljurf M Mohty M Almohareb F Ahmed SOA . An update on the molecular pathogenesis and potential therapeutic targeting of AML with t(8;21)(q22;q22.1);RUNX1-RUNX1T1. Blood Adv. (2020) 4:229–38. doi: 10.1182/bloodadvances.2019000168, PMID: 31935293 PMC6960481

[23] Faber ZJ Chen X Gedman AL Boggs K Cheng J Ma J . The genomic landscape of core-binding factor acute myeloid leukemias. Nat Genet. (2016) 48:1551–6. doi: 10.1038/ng.3709, PMID: 27798625 PMC5508996

[24] Thomas ME 3rd Qi W Walsh MP Ma J Westover T Abdelhamed S . Functional characterization of cooperating MGA mutations in RUNX1::RUNX1T1 acute myeloid leukemia. Leukemia. (2024) 38:991–1002. doi: 10.1038/s41375-024-02193-y, PMID: 38454121 PMC11073986

[25] Huang G Cai X Li D . Significance of targeting DNMT3A mutations in AML. Ann Hematol. (2025) 104:1399–414. doi: 10.1007/s00277-024-05885-8, PMID: 39078434 PMC12031811

[26] Wang B Wen L Wang Z Chen S Qiu H . Differential implications of CSF3R mutations in t(8;21) and CEBPA double mutated acute myeloid leukemia. Clin Lymphoma Myeloma Leuk. (2022) 22:393–404. doi: 10.1016/j.clml.2021.11.013, PMID: 34975010

[27] Bark SA Dalmolin M Malafaia O Roesler R Fernandes MAC Isolan GR . Gene expression of CSF3R/CD114 is associated with poorer patient survival in glioma. Int J Mol Sci. (2024) 25:3020. doi: 10.3390/ijms25053020, PMID: 38474265 PMC10931759

[28] Arfelli VC Schmitz W Kirmaier ME Monte ER Kerbs P Cusan M . ZBTB7A loss promotes synthesis of lipids and ketone bodies in myeloid leukemia. Blood. (2023) 142:2. doi: 10.1182/blood-2023-185682

[29] Wu SY Short NJ Nasr L Dabaja BS Fang PQ . Central nervous system prophylaxis and treatment in acute leukemias. Curr Treat Options Oncol. (2022) 23:1829–44. doi: 10.1007/s11864-022-01032-5, PMID: 36510037 PMC9767998

